# Online-CBT for endometriosis: Exploring the acceptability and impact of the Ed.iTh program

**DOI:** 10.1016/j.invent.2026.100906

**Published:** 2026-01-24

**Authors:** Carola Hajek Gross, Jette Angenendt, Elena Strobl, Kathrin Schubert, Cornelia Weise

**Affiliations:** a Philipps-Universität Marburg, Dept. of Psychology, Clinical Psychology and Psychotherapy, Gutenbergstraße 18, 35032, Marburg, Germany; b Friedrich-Alexander-Universität Erlangen-Nürnberg, Dept. of Psychology, Clinical Psychology and Behavioral Health Technology, Nägelsbachstr. 49b, 91052, Erlangen, Germany

**Keywords:** CBT, Endometriosis, Women's health, Internet-based intervention

## Abstract

**Theoretical background:**

Psychological distress significantly contributes to impairment in endometriosis. However, patients face systemic barriers to psychological support, including long waiting times, stigma, and a lack of endometriosis-specific expertise. Internet-based CBT (iCBT) offers a promising alternative, yet its acceptability and perceived helpfulness in addressing the psychological needs of endometriosis patients remains unexplored.

**Aim:**

This study aimed to evaluate users' experiences with Ed.iTh, the first therapist-guided iCBT program developed for individuals with endometriosis. The program includes psychoeducation, cognitive restructuring, pacing, emotion regulation, and asynchronous therapist support.

**Methods:**

*N* = 163 individuals with endometriosis were invited to complete the eight-week Ed.iTh program. Each week, users engaged with a new module and provided written feedback on (1) the content and (2) suggestions for improvement. Free-text responses (1678 statements from 137 users) were analyzed using directed content analysis, based on the DeLone and McLean model (2003), which evaluates the success of information systems (e.g., internet-based interventions) across six dimensions.

**Results:**

Participants expressed overall satisfaction with the program. They appreciated the relevance and clarity of the provided information (information quality) and emotional support, which helped reduce feelings of isolation (individual impact). Some participants noted difficulties applying techniques independently and requested more concrete guidance (service quality). Others highlighted a need for more interactivity and flexible pacing (system quality).

**Discussion:**

The findings demonstrate the acceptability of Ed.iTh and highlight the potential of iCBT as a low-threshold treatment option for endometriosis. Suggested improvements include greater personalization, more flexible scheduling, and enhanced interactivity to increase engagement.

## Highlights

• First therapist-guided iCBT tailored to endometriosis was evaluated.
• 1678 user comments analyzed using DeLone & McLean IS success model.
• Biopsychosocial content helped participants feel validated and less alone.
• Participants suggested improvements to pacing, personalization and scaffolding.
• Feedback indicates feasibility and acceptability of iCBT in this population.

## 1 Introduction

Endometriosis is a systemic condition, characterized by the growth of endometrial-like tissue outside the uterine cavity, affecting approximately 5–10% of reproductive-aged individuals with a uterus (Taylor et al., 2021). In addition to hallmark symptoms such as dysmenorrhea, dyspareunia, chronic pelvic pain, and infertility (Mechsner, 2016), endometriosis imposes a substantial psychological and functional burden. Compared to healthy controls, individuals with endometriosis experience significantly elevated symptoms of depression (SMD = 0.71; 95% CI, 0.36–1.06) and anxiety (SMD = 0.60; 95% CI, 0.35–0.84) (Van Barneveld et al., 2022). Psychological distress stems not only from pain but also from a loss of control over daily life, uncertainty about disease progression and fertility, and diminished self-esteem (Moradi et al., 2014). Qualitative research further highlights the social impact of endometriosis such as strained relationships and reduced participation in daily life (Facchin et al., 2021; Rush and Misajon, 2018). Notably, emotional distress is a key predictor of long-term impairment in endometriosis (Dowding et al., 2023), while functional limitations drive significant productivity losses and societal costs (Heinze et al., 2024).

Given the wide-ranging impacts of endometriosis, there is growing recognition of the need for psychologically informed care that goes beyond standard biomedical treatment, which often fails to provide sustained symptom relief (Becker et al., 2017; Van Niekerk et al., 2019). Cognitive-behavioral therapy (CBT) offers a complementary approach by targeting the biopsychosocial factors that shape pain perception and emotional functioning, equipping individuals with strategies to regain control despite persistent symptoms (Turk et al., 2008). CBT is an evidence-based intervention for chronic pain (Williams et al., 2020; Yang et al., 2022), and emerging findings suggest that CBT-based interventions may also benefit individuals with endometriosis by reducing pain, improving psychological outcomes, and enhancing quality of life, though more rigorous trials are needed to strengthen the evidence base (Donatti et al., 2022).

While clinical guidelines recommend integrating psychotherapy into multimodal endometriosis care (Becker et al., 2022; Burghaus et al., 2021), multiple barriers continue to limit access to psychological treatment. At the institutional level, challenges include insufficient treatment capacity, delayed referrals, and limited provider expertise regarding the psychological dimensions of endometriosis (Boersen et al., 2021; Dowding et al., 2024). At the patient level, hesitation to seek therapy may stem from fear of stigmatization or misconceptions about the value of psychological interventions in treating chronic pain (Boersen et al., 2021). Practical constraints, such as long travel distances, financial burdens, and time demands, further hinder access (Boersen et al., 2021; Dowding et al., 2024). As a result, many individuals who could benefit from psychological support remain underserved.

Internet-based CBT (iCBT) offers an evidence-based alternative for individuals who cannot or prefer not to seek face-to-face therapy. Meta-analyses indicate that iCBT can produce outcomes comparable to face-to-face therapy across various conditions, including chronic pain (Terpstra et al., 2022), with sustained effects at follow-up observed in conditions such as depression, anxiety and chronic fatigue (Andersson et al., 2018). While no iCBT program has yet been evaluated specifically for endometriosis, emerging digital initiatives such as the German EndoApp demonstrate the value of delivering condition-specific education and self-management tools (e.g. relaxation exercises) via mobile platforms (Zugaj et al., 2024). However, such self-guided tools are not designed to invoke a structured psychotherapeutic process. Therapist-guided iCBT, by contrast, integrates core CBT strategies (e.g. cognitive restructuring, pacing, and emotion regulation) into modular, interactive formats reinforced by personalized therapist feedback. This approach holds promise for addressing entrenched behavioral and cognitive patterns in endometriosis populations, such as avoidance and catastrophizing (González-Echevarría et al., 2019; Zarbo et al., 2018), and may extend digital care to individuals with more persistent clinical symptoms.

Despite the clinical potential of therapist-guided iCBT, its acceptability in individuals with endometriosis remains to be established. Preliminary findings from a focus group study suggest that endometriosis patients (*N* = 17) have concerns about whether web-based CBT, particularly in the absence of synchronous interaction, can provide the emotional support necessary, raising important questions about how therapist contact should be delivered in a digital context to foster a sense of being “seen and heard” (Boersen et al., 2021). Beyond emotional support, additional challenges associated with endometriosis, such as fatigue that can impair cognitive functioning (Horn et al., 2025) and heterogeneous symptom presentations (Mitchell et al., 2024), may complicate engagement in a digital format and lead to varying levels of expectations and responsiveness to iCBT. These challenges raise critical questions about how therapist-guided digital interventions need to be designed to address the specific needs of individuals with endometriosis and serve as a viable alternative to in-person therapy.

The present study aims to address this gap by exploring users' experiences with Ed.iTh, the first therapist-guided iCBT program specifically developed for individuals with endometriosis (Schubert et al., 2022). A qualitative approach was chosen, as it is well-suited to uncover nuanced barriers and facilitators that may affect adherence and outcomes in digital health interventions (Kaltenthaler et al., 2008). Guided by the updated DeLone and McLean (D&M) model of information systems success (Delone and McLean, 2003), previously applied to evaluate an iCBT intervention (Johnsen and Haddeland, 2021), the study examines six core dimensions of the intervention: information quality, system quality, service quality, use, user satisfaction, and individual impact. This framework enables a comprehensive assessment of both structural and experiential aspects of the program, with the aim to inform the design of future digital interventions for endometriosis.

## 2 Methods

### 2.1 The iCBT intervention

‘Ed.iTh’ is a therapist-guided iCBT intervention aiming to improve the quality of life of individuals with endometriosis. It was developed through a participatory process involving experts in psychology, medicine, and gender studies, alongside direct input from individuals with endometriosis. Grounded in CBT principles, the intervention comprises eight modules covering: the biopsychosocial model, cognitive restructuring, stress and pain management, emotion regulation, communication (e.g., with healthcare providers, employers), and goal maintenance (see Table 1). Participants complete one module per week and spend an estimated 1–2 h on each module. Modules include reflection questions and practical exercises to reinforce key concepts. Therapist support is provided through weekly written feedback on these interactive elements and the option to message the assigned therapist for clarification or assistance. All therapists hold at least a bachelor's degree in psychology and are supervised by a licensed CBT therapist. Each module features fictional personas with endometriosis to enhance relatability. Engagement is supported through audio relaxation and meditation exercises, videos, and animations. Worksheets for cognitive restructuring and activity scheduling are provided and participants can download their notes on all modules upon program completion. Ed.iTh is delivered via an online platform designed for computer use.

**Table 1:** Overview of module content in the Ed.iTh iCBT program.

Week	Module name	Module content
1	Module 1: Getting started with the training	Psychoeducation, goal setting
2	Module 2: Thoughts I—Our constant companions	Cognitive restructuring I—Using the ABC model
3	Module 3: Thoughts II—Who controls whom?	Cognitive restructuring II—Using the ABCD model
4	Module 4: My pain and me—And still I want to do something	Dealing with acute pain, pacing, weekly schedule
5	Module 5: Stress and pain—Always everything at once	Stress management (recovery skills, dealing with acute stress, contingency plans)
6	Module 6: Emotions—Accepting and tolerating	Psychoeducation, accepting and regulating negative emotions, mindfulness
7	Module 7: Clear communication	Basic communication rules, flexible handling of different situations (e.g. sexuality)
8	Module 8: My plan for the future	Summary, setting of long-term goals

### 2.2 Study design and data collection

This qualitative study was embedded in a randomized controlled trial (RCT) evaluating the efficacy of Ed.iTh for improving health-related quality of life. In the trial, *N* = 163 participants were randomized to either guided iCBT (*n* = 82) or a waitlist control group (*n* = 81), which received the intervention after completing a three-month follow-up. Further details are available in the study protocol (Schubert et al., 2022).

**Fig. 1:**
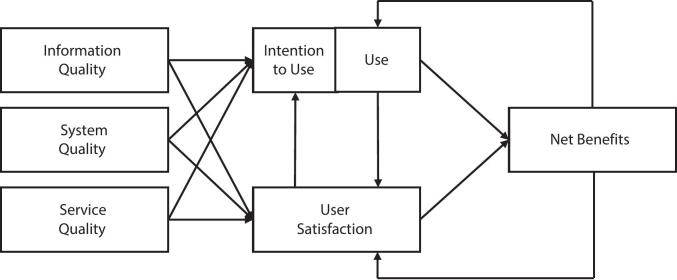
The updated DeLone and McLean IS success model (2003).

To examine participants' experiences with the intervention, we analyzed written feedback provided at the end of each module. Participants responded to two open-ended questions: (a) *What content did you find helpful?* and (b) *Do you have any suggestions for improvement?.* We conducted a directed content analysis (Hsieh and Shannon, 2005) to systematically assess participants' responses, using the updated D&M model (2003) as our theoretical framework (see Fig. 1). Our research approach was inspired by Johnsen and Haddeland (2021), who first applied the D&M model to qualitatively evaluate a guided iCBT for anxiety disorders. The D&M model is a well-established framework traditionally used to assess the effectiveness of information systems, such as e-government systems, online communities, and social networks (Jeyaraj, 2020). It evaluates interventions across six dimensions: Information Quality, System Quality, Service Quality, Use, User Satisfaction, and Net Benefits. According to DeLone and McLean (2003), these six dimensions are interrelated and interact dynamically. For example, when an intervention provides high-quality content, intuitive usability, and effective support, it can foster user satisfaction and promote sustained engagement. As users increasingly perceive individual benefits, their continued use and satisfaction further reinforce one another, ultimately enhancing the intervention's overall success (DeLone and McLean, 2003).

(*Note*. Adapted from *The DeLone and McLean Model of Information Systems Success: A Ten-Year Update*, by W. H. DeLone and E. R. McLean, 2003, *Journal of Management Information Systems, 19*(4), p. 10 (doi:https://doi.org/10.1080/07421222.2003.11045748). Copyright 2003 by Taylor & Francis.)

In the present study, we applied the D&M model (2003) post hoc as a coding framework to structure the analysis of participant feedback. The six core dimensions served as overarching coding categories. To capture context-specific features of iCBT, we adapted subcategories identified by Johnsen and Haddeland (2021) in their qualitative iCBT evaluation. These subcategories (e.g. “relevance of content”, “ease of understanding”) reflect applied indicators of each dimension (e.g. information quality) based on prior model adaptations (Petter et al., 2008). Following the approach of Johnsen and Haddeland (2021), we relabeled the original *Net Benefits* dimension as *Individual Impact* to better reflect the patient-centered outcome of this psychological intervention. This approach aligns with DeLone and McLean's (2003) emphasis on tailoring the model to the specific evaluation context (e.g. iCBT), while maintaining alignment with previous applications of the framework. Table 2 provides an overview of the six dimensions and corresponding subcategories in this study.

**Table 2:** Coded dimensions of the D&M IS success model with subcategories and exemplary participant quotes.

Dimensions of the D&M IS success model (2003)	Subcategories adapted from Johnsen and Haddeland (2021)	Exemplary participant quotes
1.Information quality	Ease of understanding	“It was particularly helpful for me that the topic of sexuality and the problems associated with endometriosis were discussed in such detail”
	Relevance of content	
	*Completeness	
	*Conciseness	
2.System quality	Ease of use	“It would be more helpful if the processing period of a lesson were longer. In addition to my job, everyday life and the unplannable endometriosis-related downtime, I would like to take my time and work intensively on possible strategies”
	System flexibility	
	Desirable characteristics	
3.Service quality	Information support	“It's nice that someone gets in touch every week in writing, but somehow it would also be nice to just get a phone call.”
	Follow-up and guidance	
	**Introduction to the program	
4.Use	Attitude towards using and re-using the program	“Pacing was particularly helpful for me. I would like to try it out”
	Patterns of usage	
5.Satisfaction	Opinions	“Thank you for allowing me to take part in this training and I very much hope that it will be made available to many people affected.”
	Perceived advantages and disadvantages	
6.Individual Impacts	Gain of knowledge	“I don't know whether my pain has actually decreased, but I certainly perceive it as less dominant.”
	Competence to cope	

*Note.* *added inductively based on participant feedback **not identified in the present data.

### 2.3 Coding and analysis procedure

All written feedback was uploaded to MAXQDA 24 (VERBI Software, 2023) by the first author (CHG). A coding matrix was developed based on the six main categories of the D&M model (2003) and the subcategories identified by Johnsen and Haddeland (2021). The coding matrix was shared with a second coder (JA). To pilot the coding matrix, both coders independently coded feedback from the first module and subsequently discussed their interpretations and any discrepancies to ensure consistency. Subsequently, participant responses from the remaining modules were analyzed. Any discrepancies were resolved through discussions. When participant responses could be assigned to multiple categories, it was placed in the category that provided the best conceptual fit. Consensus was reached in all cases. Responses that did not align with the predefined subcategories were flagged for inductive coding. As a result, two new subcategories, “*Completeness*” and “*Conciseness*”, were added under the dimension *Information Quality*. One original subcategory from Johnsen and Haddeland's (2021) framework, *“Introduction to the Program”*, was not identified in the data and was excluded. As a final step, the first author reviewed the text within each subcategory to synthesize findings for the analysis, which were then shared and discussed with the second coder. In total, 1682 coded statements were extracted from responses provided by *N* = 137 participants. Four statements were excluded as they did not provide feedback on the Ed.iTh program (e.g. comments on the RCT questionnaire). Representative quotes in the results section were translated from German to English while ensuring the preservation of their original meaning.

### 2.4 Participant recruitment

Participants were individuals diagnosed with endometriosis, recruited between October 2022 and June 2023 through online platforms, mailing lists, self-help groups, and referrals from local clinics and gynecologists. Inclusion criteria were age between 18 and 45, laparoscopically confirmed endometriosis, and reduced health-related quality of life, defined as a total summed raw score of ≥15 across all items of the Endometriosis Health Profile-30 plus 23-item modular section (EHP-30; Jones et al., 2001), noting that no clinically validated cut-off exists for this instrument. Participants were also required to have sufficient German language skills and access to a stable internet connection and a suitable device (PC or laptop). Exclusion criteria included severe depression, bipolar or psychotic disorders, and substance use disorder, assessed via the Web-Based Screening Questionnaire (WSQ; Donker et al., 2009), a telephone interview and the Beck Depression Inventory-II (BDI-II; Beck et al., 1996). Participants were also excluded if they had recently participated in CBT, had undergone a hysterectomy, or regularly used benzodiazepines. Participants with changes in antidepressant or hormonal contraceptive use within the past three months were able to reach out again when their medication (substance and dosage) had remained stable and were then, if meeting all other criteria, included in the study. Individuals undergoing or planning fertility treatment (e.g., IUI, IVF, ICSI with hormonal stimulation), those who were pregnant, postpartum, or breastfeeding within the last six months, and individuals with medical conditions (e.g., malignant tumors, inflammatory bowel diseases, or specific viral infections) were also excluded. Informed consent was obtained after providing detailed study information. Of the 329 individuals expressing interest in the study, 163 met eligibility criteria and were randomized. Detailed participant flow and reasons for exclusion are described in Supplementary Material (S1).

### 2.5 Ethics

This study was approved by the Ethics Committee of the Department of Psychology at the Philipps University Marburg (approval number: 2021-45v).

## 3 Results

**Fig. 2:**
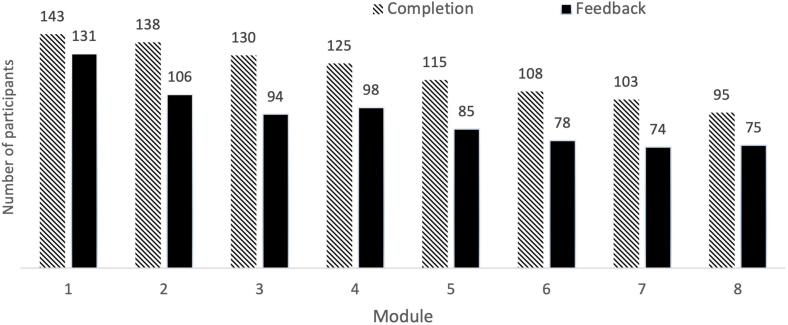
Participants' completion and feedback across Ed.iTh modules (*N* = 163).

A total of *N* = 137 participants provided written feedback on at least one module. Their demographic and clinical characteristics are summarized in Table 3. Fig. 2 illustrates the number of participants from the original RCT who completed each module and how many provided feedback at each stage. Of the 137 participants who provided qualitative feedback, 44 (32%) commented on all eight modules, 20 (15%) on seven, 13 (9%) on six, 11 (8%) on five, 10 (7%) on four, 10 (7%) on three, 17 (12%) on two, and 12 (9%) on one module. The average number of statements per participant ranged from 2.0 to 2.6 statements per module (Module 1: 2.4, Module 2: 2.1, Module 3: 2.2, Module 4: 2.6, Module 5: 2.1, Module 6: 2.2, Module 7: 2.3, Module 8: 2.0). Among the 143 participants who completed at least one module, most participants (69%, *n* = 99) provided feedback on the majority of modules they completed (75–100%), 21 participants (15%) provided feedback on 50–74% of completed modules, 17 participants (12%) on 25–49%, and six participants (4%) provided feedback on 0–24% of modules despite completing them.

**Table 3:** Participant characteristics (*N* = 137).

Characteristic	
Mean age, years ± SD, years	30.7 ± 5.9
Family status	
Single, *n* (%)	36 (26.3)
Stable relationship, *n* (%)	69 (50.4)
Married/registered partnership, *n* (%)	31 (22.6)
Divorced, *n* (%)	1 (0.7)
Highest level of education	
Secondary school, *n* (%)	3 (2.2)
Further education, *n* (%)	23 (16.8)
Vocational training*, n* (%)	31 (22.6)
University degree, *n* (%)	77 (56.2)
Doctoral degree, *n* (%)	3 (2.2)
Employment status	
Student, *n* (%)	22 (16.1)
Apprentice, *n* (%)	4 (2.9)
Employed, *n* (%)	91 (66.4)
Self-employed*, n* (%)	5 (3.6)
Unemployed*, n* (%)	1 (0.7)
Other, *n* (%)	14 (10.2)
Children	
0, *n* (%)	119 (86.9)
1, *n* (%)	10 (7.3)
2, *n* (%)	8 (5.8)
Medical characteristics	
Menstruation, *n* (%)^a^	76 (55.5)
Mean pain during menstruation ± SD (1−10)	6.9 ± 1.9
Mean time since laparoscopy ± SD, years	3.1 ± 3.1
Endometriosis treatment	
Surgical removal, *n* (%)^a^	132 (96.4)
Psychotherapy, *n* (%)^a^	22 (16.1)
Nutritional counseling, *n* (%)^a^	31 (22.6)
Physiotherapy, *n* (%)^a^	37 (27.0)
TENS, *n* (%)^a^	16 (11.7)
Homeopathy, *n* (%)^a^	26 (19.0)
Osteopathy, *n* (%)^a^	54 (39.4)
TCM, *n* (%)^a^	20 (14.6)
Contraceptives, *n* (%)^a^	101 (73.7)
Pain killers, *n* (%)^a^	75 (54.7)
Psychological characteristics	
Mean BDI-II ± SD (0–63)	18.5 ± 10.3
Mean EHP-30 ± SD (0–100)	48.4 ± 18.1
Participation in the ‘Ed.iTh’ program	
Completion, *n* (%)	93 (67.9)

*Note.* EHP-30 = Endometriosis-related Quality of Life; Surgical removal: Surgical removal of the endometriosis (ablation or excision), e.g., during a laparoscopy; TENS: Transcutaneous electrical nerve stimulation for pain relief; TCM: Traditional Chinese Medicine; BDI-II = Beck Depression Inventory-II. ^a^ Reflects the number and percentage of participants answering “yes” to this question.

To evaluate participants' experience with the Ed.iTh program, the D&M model (2003) was applied. The results are presented according to its six dimensions: Information Quality, System Quality, Service Quality, Use, User Satisfaction, and Individual Impact. For each dimension, subcategory, and theme, we report the number of participants (*n*) who provided feedback and the total number of statements they contributed. When participants each contributed exactly one statement to a theme, we report only the single number.

### 3.1 Information quality (*n* = 130; 473 statements)

The dimension “Information Quality” assesses how relevant, comprehensive, and clear the content is in supporting users to achieve their therapeutic goals.

#### 3.1.1 Relevance of content (*n* = 107; 281 statements)

Content deemed most relevant by participants included balancing activity levels (*n* = 54; 67 statements), identifying cognitive distortions (*n* = 41; 47 statements), improving communication and self-advocacy (*n* = 27; 28 statements), pain education (*n* = 21; 27 statements), emotional processing (*n* = 24), and reflecting on the biopsychosocial impact of endometriosis (*n* = 12). Twenty-one valued the program's content on sexuality, noting that it is often neglected in medical care. One participant described the content on sexuality as “extremely important, de-tabooing and helpful” (P1). Fifteen appreciated the examples used in the lessons, describing them as relatable, e.g., “Thank you for these ‘perfectly fitting’ examples! It seemed as if you had ‘cut them right out of me’” (P2). Fourteen found the introductory information on endometriosis unnecessary due to their prior knowledge, although five acknowledged its value for those newly diagnosed. Twelve (15 statements) viewed the pain management and pacing content as less relevant due to pain relief following surgery or hormonal treatment, while two expressed a wish for this content to appear earlier in the program. Eight (9 statements) suggested offering flexible paths so users could choose content based on their personal needs.

#### 3.1.2 Ease of understanding (*n* = 58; 65 statements)

Nine participants (12 statements) found that the provided examples enhanced their understanding, e.g., “[the examples] are great for understanding things that are initially unclear, and I can relate to many” (P3). Eighteen (23 statements) indicated a desire for additional examples. Furthermore, fifteen reported difficulties grasping certain contents, particularly the ABC model and pacing. One participant shared that they “still didn't understand the ABC model at the third attempt” (P4), attributing this to unclear explanations and the absence of a supporting video. Eleven (15 statements) expressed confusion about the purpose of certain exercises. For instance, one noted, “I'm not 100% aware of how exactly knowing about the connections between thoughts, feelings, and behavior helps me with endometriosis” (P5).

#### 3.1.3 Conciseness (*n* = 26; 29 statements)

Six participants were satisfied with the lesson length, while seventeen (18 statements) described them as “too lengthy” or “dense”. Six expressed frustrations that lessons took more time to complete than they were willing to dedicate, often requiring two to four hours. One noted: “I have to admit that my frustration is very high right now. The lessons are taking up too much time... I think the lessons are good, it's not like that, but... four hours a week is just too much for me” (P6). Two felt that the high content density made the program feel like an obligation rather than something they wanted to engage with. Several suggested dividing the more extensive modules to allow for more mindful engagement. For example, five would have preferred the relaxation techniques to be spread across multiple sessions. As one noted: “That way, the exercises would be less condensed, and I would be motivated to do them every week” (P7).

#### 3.1.4 Completeness (*n* = 63; 98 statements)

Several participants requested more in-depth coverage of specific topics. Commonly mentioned were the biopsychosocial impacts of endometriosis (*n* = 14), the biology of pain and its chronification (*n* = 7), and sexuality and partnership (*n* = 7). Others wished for expanded content on cognitive distortions and the ABC model (*n* = 6), relaxation or mindfulness exercises (*n* = 4), emotional coping (*n* = 3), and activity pacing (*n* = 1). Some requests emerged for topics covered in later modules, such as managing negative thoughts (*n* = 13), pain coping strategies (*n* = 7), and emotion regulation (*n* = 1). Participants also suggested adding new topics, including medical and alternative treatments (*n* = 13; 14 statements), nutrition (*n* = 9; 12 statements), adenomyosis (*n* = 3), fatigue (*n* = 3), infertility (*n* = 2), and patient rights (*n* = 1).

### 3.2 System quality (*n* = 106; 311 statements)

The dimension “System Quality” assesses whether the platform provides a seamless, engaging user experience.

#### 3.2.1 Ease of use (*n* = 8; 8 statements)

Only five participants reported frustration with platform navigation. Three encountered issues with unsaved edits and had to re-enter responses. One noted: “I had to edit part of the lesson twice and my detailed answers were lost – annoying” (P8). Two wished for better mobile usability due to difficulties editing on phones. Suggestions included adding a progress-tracking sidebar (*n* = 1), expanding the size of free-text fields (*n* = 1), and incorporating more multiple-choice questions to reduce writing (*n* = 1).

#### 3.2.2 System flexibility (*n* = 25; 26 statements)

Eighteen participants wished for more time per module or greater scheduling flexibility, finding the weekly pace too fast to fully engage with the material. One noted: “These are all such important things, but I just think that it is all happening way too fast” (P9). Three described feeling stressed when unable to meet their own engagement standards. As one put it: “I would like to take my time and work intensively on possible strategies. For me personally, this is not possible to my satisfaction in the short time available and creates additional stress” (P10). Five advocated for longer intervals between lessons (e.g., two to three weeks), two wished for a pause-and-resume option, and one requested upfront access to the entire course. Several participants cited personal reasons for needing more time, such as endometriosis-related downtime or work demands. Seven (8 statements) suggested that knowing the upcoming module's exercises in advance would help them allocate time to complete them during the week.

#### 3.2.3 Desirable characteristics (*n* = 101; 277 statements)

Forty-seven participants appreciated receiving a compiled summary of their written lesson entries at the end of the intervention, while thirty-three (38 statements) expressed a wish to download lesson content throughout the course, for example in form of a workbook. Thirty-eight (46 statements) highlighted that videos, audio files, and graphics enhanced engagement and emotional connection: “The video particularly touched me” (P11); “It's great that audio was also included” (P12). Fifteen (19 statements) requested more multimedia, including videos featuring individuals affected by endometriosis. Feedback on the audio files included concerns about loud background music (*n* = 6), inconsistent tone (*n* = 4), requests for downloadable audio files (*n* = 4) and a preference for more soothing or male voices (*n* = 2). Free-text fields (*n =* 20; 23 statements) were valued for encouraging self-reflection and consolidation: “It helps with processing the story and internalizing the content” (P13). Practical exercises were also highly appreciated (*n* = 40; 53 statements); seventeen requested additional exercises, particularly related to the ABC model and emotional regulation. Five valued downloadable worksheets provided in some lessons (e.g., for cognitive restructuring), while six wished for additional worksheets. Eight (9 statements) found lessons overly text-heavy: “Less text, sometimes it's too exhausting” (P12). Two requested a feature to highlight key-takeaways on the platform.

### 3.3 Service quality (*n* = 33; 38 statements)

The dimension “Service Quality” assesses the adequacy of therapist support and its contribution to a positive user experience.

#### 3.3.1 Information support (*n* = 29; 34 statements)

Twenty-nine participants commented on the received therapist support. Ten praised the individualized feedback for being personally attuned and clarifying key concepts: “The personal emails are always perfect, they pick me up exactly where I need them” (P2); “thanks to the feedback I was able to revise my ABC models much more easily” (P14). Fourteen (15 statements) wanted more support, especially for applying theoretical models like ABC: “More help for finding a situation would be good” (P15). Only two reported contacting their therapist directly, both found this helpful. Six (9 statements) desired more personal, face-to-face interactions, e.g., “It would be more useful to discuss things in person” (P16).

#### 3.3.2 Follow-up and guidance (*n* = 4; 4 statements)

Only three commented on desiring follow-up communication and further resources. One participant mentioned, “I would be delighted to receive a final message from [accompanying therapist]. And maybe again after a few weeks to have a bit of support” (P17). Another participant wished for a few more guided exercises after finishing the intervention. One expressed interest in participating in future studies, noting, “I hope that you will give me the chance to take part in your studies again” (P18).

### 3.4 Use (*n* = 56; 84 statements)

The dimension “Use” assesses the extent to which participants intend to and actually engage with the program.

#### 3.4.1 Attitudes towards using and reusing the program (*n* = 41; 58 statements)

Thirty-four (44 statements) reported intentions to apply specific strategies from the intervention in daily life, e.g., “I find the pacing interesting and I will make sure to do it more often” (P19), “I should definitely keep trying [the ‘Let-it-be’ list]” (P20). Four emphasized that techniques like the ABC model required repetition for long-term benefit. Six found the online format a barrier: “I was satisfied with the lesson, but I don't think online training is really for me” (P21). Three (4 statements) wished for peer exchange: “it would help me a lot more to discuss things online in a group” (P22).

#### 3.4.2 Patterns of use (*n* = 23; 26 statements)

Seven participants appreciated dedicating weekly time to focus on their illness, e.g., “The continuity of the program is very helpful… I now actively take the time once a week to deal with my illness” (P23). Thirteen (14 statements) reported difficulties implementing the exercises, often due to a lack of time or forgetfulness, and requested more practical tips: “I find it very difficult to implement the exercises… more tips on making it easier would be helpful” (P24). Five had trouble staying focused or completing tasks due to health or work-related challenges.

### 3.5 User satisfaction (*n* = 122; 402 statements)

The dimension “User satisfaction” assesses overall attitude towards and contentment with the program.

#### 3.5.1 Opinions (*n* = 115; 365 statements)

Satisfaction with the program was reflected in 358 statements (*n* = 113), including 27 (*n* = 23) that conveyed high praise, e.g., “The way it is explained, the examples, the direct reference to Endo really makes the study outstanding” (P2), and 101 statements (*n* = 54) reflecting no wishes for improvement. Several appreciated the considerate language and thoughtful design, with participants expressing hope that the program would be made widely available. Seven voiced dissatisfactions, mainly due to the focus on adapting to endometriosis, e.g. “It bothered me that the program suggested integrating endometriosis into life. I believe that with psychological help, it can be overcome” (P26). Another described the images of “young, attractive, thin women” on the platform as disempowering (P27).

#### 3.5.2 Perceived advantages and disadvantages (*n* = 29; 37 statements)

Perceived advantages of the intervention included reduced feelings of isolation, reassurance and validation. Fourteen (17 statements) found comfort in realizing they were not alone: “It gave me the feeling that others feel the same way and that I'm not alone with my thoughts/problems” (P28). Eleven (13 statements) felt reassured that symptoms like fatigue, mental health issues, or sexual difficulties were legitimate: “It was important for me to read again in black and white that all pain is real” (P29). Another appreciated the broader scope on endometriosis beyond pain. Six found emotional reflection taxing: “It was more emotionally exhausting than I thought to confront my thoughts and feelings” (P30).

### 3.6 Individual impacts (*n* = 115; 370 statements)

The dimension “Individual Impacts” assess the outcomes resulting from the use of the program.

#### 3.6.1 Gain of knowledge (*n* = 108; 283 statements)

Participants frequently highlighted gains in knowledge, insight, and coping strategies. Most (*n* = 74; 99 statements) valued the ABC model for shifting perspectives and managing emotions. One shared: “Becoming aware of my thoughts and feelings was very good and important. I tend to ignore what is going on inside me” (P31). Thirty-seven (38 statements) reflected on emotions and unmet needs: “The realization that there is an unmet need behind every emotion helps me better understand and communicate my feelings” (P32). Thirty-four (37 statements) emphasized the importance of balancing over- and under-activity using tools like pacing or the Let-it-be list: “The pacing concept made me aware of how to test my limits without overloading myself, which is crucial for managing my condition” (P33). Thirty-two gained awareness of how endometriosis affects their lives: “It became clear to me which areas of my life are affected, and that I can do a lot for my well-being” (P34). Others reported improved communication (*n* = 30), empowerment through pain-coping strategies (*n* = 18), relaxation and mindfulness routines (*n* = 17), and understanding of the connection between stress and pain (*n* = 12).

#### 3.6.2 Competence to cope (*n* = 57; 87 statements)

Twelve participants expressed that their coping had improved by the end of the program. One wrote: “I feel I have truly learned a better way of dealing with endometriosis” (P35). Thirty-two (37 statements) reported increased optimism and self-efficacy, recognizing their ability to influence their circumstances: “This online therapy finally showed me that there is another way and that you can change your perspective with great exercises” (P36). Twenty-eight described positive effects from applying coping strategies in everyday life. For example, one noted the ABC model had a “soothing effect” (P7), while another perceived reduced pain and shared how great it felt “to see what is possible in eight or nine weeks” (P37). Eight (10 statements) expressed doubts about specific strategies, with one finding the ABC model “too disembodied” to be helpful (P38). Only one participant questioned the long-term impact, stating, “I am unsure whether I can really handle my problems better in the long term” (P39).

## 4 Discussion

This is the first study to explore users' experiences with a therapist-guided iCBT program tailored to endometriosis. Using directed content analysis informed by the D&M model (2003), the findings reveal positive resonance, particularly in terms of information quality, user satisfaction, and individual impact. The richness of participant feedback indicates that users engaged thoughtfully with both the intervention and its evaluation, while also highlighting concrete opportunities for design improvements of digital interventions for this population.

### 4.1 Information quality

One of the key strengths of the Ed.iTh program was the overall relevance of the provided information, aligning with the D&M model (2003), which emphasizes the importance of information quality in driving user satisfaction and outcomes. Endometriosis patients often struggle with the emotional burden of unpredictable symptoms, impairments in daily activities, and feelings of invalidation, all of which contribute to a sense of loss of control (Moradi et al., 2014). Given these challenges, it is not surprising that participants particularly valued actionable CBT techniques aimed at restoring agency (Turk et al., 2008), such as pacing for pain management and cognitive restructuring to address emotional distress. Our findings align with emerging research highlighting the value of condition-specific education and self-management strategies delivered digitally for endometriosis patients (Zugaj et al., 2024). At the same time, we extend this knowledge by demonstrating that structured CBT content can be delivered online and is well-accepted by this population, supporting the expansion of digital psychological care for individuals with endometriosis beyond psychoeducation. Although participants appreciated the program's biopsychosocial framing, they also called for greater customization of modules to account for symptom diversity (Mitchell et al., 2024) and varying levels of prior knowledge. Evidence from iCBT for depression suggests user-directed content selection may lead to more meaningful improvements than clinician-directed modules (Andersson et al., 2023). Adopting similar flexibility in Ed.iTh could allow users to prioritize relevant topics and avoid redundancy. Despite these suggestions, the program's content resonated with a broad range of users, supporting its potential for wider dissemination.

### 4.2 System quality

The evaluation of the Ed.iTh program revealed both strengths and barriers in system quality, highlighting the role of system quality in shaping user satisfaction and engagement. Self-reflection exercises were highly valued for facilitating deeper emotional insight, highlighting a preference among endometriosis patients for active engagement over passive content consumption. Multimedia elements, known to reduce cognitive load and enhance learning in digital environments (Lackmann et al., 2021), were similarly appreciated for making lessons more engaging and digestible. Despite these strengths, participants noted barriers to engagement, consistent with previous iCBT interventions, including the limited time provided by the one-lesson-per-week schedule (Asplund et al., 2019), the length of some lessons (Fleischmann et al., 2018), the text-heavy nature of certain sections (Rozental et al., 2015) and minor technical issues (Johnsen and Haddeland, 2021). The importance of usability in digital health interventions is well-documented (Kaufman et al., 2003) and this consideration may be particularly critical for endometriosis patients, where fluctuating pain symptoms, depression and fatigue can exacerbate cognitive difficulties such as impaired concentration (Horn et al., 2025), making engagement with digital platforms more challenging. Suggestions such as shortening lessons, more multimedia, downloadable materials, and flexible scheduling have been successfully applied in prior research (Dagöö et al., 2014; Hadjistavropoulos et al., 2018; Johnsen and Haddeland, 2021) and should be considered in future versions of Ed.iTh.

### 4.3 Service quality

In a prior focus group study, Boersen et al. (2021) reported that all endometriosis participants (*N* = 17) expressed a preference for face-to-face CBT over web-based formats, highlighting the importance of feeling “seen and heard” by a therapist. Somewhat surprisingly, only 29 out of 137 participants in Ed.iTh commented on the therapeutic guidance received, suggesting that written therapist feedback may not have been a particularly salient aspect of the intervention for most users. Few participants voiced explicit concerns about the lack of personal interaction with a therapist. Instead, feedback primarily addressed practical challenges, such as difficulties in understanding or applying therapeutic concepts, which might be alleviated through clearer instructions and illustrative examples. Conversely, a subset of participants described the written therapist feedback as personally attuned and helpful in applying strategies, suggesting that for some users, guided iCBT may offer sufficient therapeutic support. This aligns with prior findings that therapeutic connection can emerge in digital interventions even without real-time interaction, particularly when support is perceived as personalized and responsive (Patel et al., 2020). It is possible that the program's condition-specific focus contributed to the perceived value of therapist input for some users, especially compared to earlier reports of frustration with therapists' limited endometriosis-specific knowledge in face-to-face settings (Dowding et al., 2024). In light of the limited feedback on therapist involvement, future research should examine whether therapist feedback offers sufficient added value to justify its resource intensity, or whether more scalable alternatives, such as on-demand support (Janse et al., 2018; Käll et al., 2023), could meet users' needs more efficiently.

### 4.4 Use

Consistent with the D&M model (Delone and McLean, 2003), which highlights the interconnectedness between information quality and use, participants reported applying techniques from Ed.iTh in their daily lives, suggesting a high degree of perceived utility of the content. Nonetheless, a subset reported difficulties applying techniques, citing challenges common to both digital and face-to-face therapy, such as competing demands (Johnsen and Haddeland, 2021; Kazantzis and Miller, 2022). Incorporating strategies like if–then planning and progress monitoring (Sheeran and Webb, 2016) may support more consistent application. Only a few participants expressed skepticism towards psychological techniques like cognitive restructuring, reflecting resistance often linked to biomedical beliefs in chronic illness contexts (Turk et al., 2008). The overall positive attitude towards utility may reflect Ed.iTh's communication, both within the program and in the study information, of how CBT might support chronic pain management (Turk et al., 2008).

### 4.5 User satisfaction

In line with the D&M (2003) model, which identifies user satisfaction as a key driver for system use, participants reported a high degree of satisfaction with Ed.iTh. Beyond valuing actionable strategies for symptom management, many underscored the significance of feeling seen and validated. This may be particularly salient in the context of endometriosis, where symptoms such as pain, fatigue, and sexual difficulties are frequently minimized or dismissed (Hudson, 2022; Requadt et al., 2024). Furthermore, participants' reports of reduced feelings of isolation throughout the intervention indicate the potential of digital interventions to buffer against the psychosocial burden associated with the condition (Calvi et al., 2024). A few participants expressed interest in peer-support features, echoing findings from the EndoApp evaluation (Zugaj et al., 2024). Given the well-established benefits of peer connection in endometriosis communities (Márki et al., 2022), the integration of forums, peer mentoring, or similar features warrants further exploration.

### 4.6 Individual impact

Feedback from Ed.iTh strengthens the growing body of literature supporting the role of CBT in improving mental health outcomes for individuals with endometriosis (Donatti et al., 2022), and demonstrates for the first time that similar psychological and behavioral gains might be achievable in a web-based format. Participants who completed the intervention described improvements in self-efficacy, coping with pain, managing daily activities, and navigating emotional challenges—domains closely tied to the functional impairment in endometriosis (Facchin et al., 2017; Moradi et al., 2014). While a randomized controlled trial is underway to evaluate Ed.iTh's efficacy (Schubert et al., 2022), our qualitative findings offer preliminary support for guided iCBT as a viable adjunct to in-person therapy. At the same time, among the 137 participants in this analysis who provided qualitative feedback, approximately one-third did not complete the intervention, underscoring the need for refinement prior to broader dissemination. System-level adjustments such as shorter lessons, more multimedia, and flexible pacing may be relevant targets for improving engagement, while requiring relatively modest additional resources. Beyond usability, the findings also emphasize that psychological care for endometriosis should include therapeutic elements that patients find particularly meaningful such as condition-specific knowledge, the inclusion of topics like sexuality, and emotional validation. Therefore, interventions like Ed.iTh may offer a model for informing psychological care across different delivery formats (Andersson, 2018).

### 4.7 Strengths and limitations

Collecting written feedback via open-ended questions following module completion minimized recall bias and captured a wide range of experiences, including from participants less likely to participate in more resource-intensive methods like focus groups or interviews. While those methods might have yielded more in-depth responses, they also risk introducing social desirability or interviewer bias. We used directed content analysis guided by the D&M model (2003), which provided a structured yet flexible framework for analyzing a large dataset. To better suit the therapeutic context, we incorporated subcategories from previous iCBT interventions (Johnsen and Haddeland, 2021). However, the feedback collected in our study was based on two open-ended questions that were not directly mapped to the D&M model's six dimensions. While this allowed participants to express themselves freely, more directive questions (e.g., “How did you perceive the guidance?”) could have elicited more detailed responses for each dimension. Despite this, the feedback aligned well with the D&M dimensions, supporting its applicability in evaluating iCBT interventions. Our large sample size (137 participants, 1678 statements) provided valuable insights, including from participants who did not complete all eight modules. Approximately 84% of participants engaged regularly by providing qualitative feedback on at least half of the modules they completed, and only 4% (*n* = 6) never provided feedback despite completing modules. However, dissatisfied participants may have provided disproportionately detailed responses and completion bias may have contributed to more positive feedback later in the program. Additionally, variability in feedback detail likely amplified stronger opinions. Furthermore, the self-selection of participants may have led to an overrepresentation of individuals already interested in digital psychological support. Of the 329 individuals who initially expressed interest, 95 (36%) were excluded based on eligibility criteria, most commonly due to ongoing hormonal treatment changes, planned or ongoing fertility treatments, recent CBT, endometriosis not being the main health burden, or a previous hysterectomy. Consequently, the sample represents a more selective subgroup of individuals with endometriosis, which may limit generalisability, as concomitant treatments and co-occurring health priorities are common in this population. Generalisability may be further limited by the high educational level of the sample, with over 50% of participants holding a university degree. A key strength of our study was that the study's qualitative approach reinforced trustworthiness criteria (Johnson et al., 2020): dependability, through consistent data collection and analysis, transferability, via detailed descriptions of the intervention and participants, and credibility, by gathering feedback directly after each module, allowing for real-time reflections. To minimize bias, coding decisions were verified through a peer debrief between two researchers, and the authors' expertise in women's mental health, CBT, and internet interventions further informed the evaluation.

### 4.8 Conclusion

There is a well-recognized need for complementary interventions in endometriosis to alleviate psychological distress. The ‘Ed.iTh’ program received favorable feedback, with participants reporting increased understanding of their condition, improved self-efficacy, and feelings of validation. These results highlight the program's potential as a low-threshold intervention that empowers individuals to manage the impacts of endometriosis more effectively. While the program's content was relevant, feedback indicates room for improvement in customization, content presentation, and support systems to facilitate the application of learned techniques in daily life. Addressing these aspects in future iterations could further enhance engagement, supporting more personalized care that aligns with the diverse needs of endometriosis patients. Practitioners may consider integrating key elements such as cognitive restructuring, stress and pain management, emotional regulation, and sexuality into therapeutic practice for a holistic treatment of endometriosis.

## CRediT authorship contribution statement

KS and CW conceived the design of the intervention and data collection. ES and JA conducted the intervention and data collection. CHG developed the analytical framework for the qualitative evaluation and prepared the data. CHG and JA coded the data. CW is the lead investigator. CHG wrote the original draft of the manuscript, which was reviewed and edited by the other authors. All authors contributed substantially to the study and approved the final manuscript.

## Declaration of Generative AI and AI-assisted technologies in the writing process

During the preparation of this work the principal author used ChatGPT-4o in order to improve the readability of the manuscript and check grammar and spelling. After using this tool, the author reviewed and edited the content as needed and takes full responsibility for the content of the publication.

## Funding statement

This study received partial funding through grants from the Outpatient Clinic for Psychotherapy, Marburg, Germany.

## Declaration of competing interest

The authors declare the following financial interests/personal relationships which may be considered as potential competing interests: Given her role on the Editorial Board of Internet Interventions, Cornelia Weise had no involvement in the peer review of this article and had no access to information regarding its peer review. Full responsibility for the editorial process for this article was delegated to another journal editor. If there are other authors, they declare that they have no known competing financial interests or personal relationships that could have appeared to influence the work reported in this paper.

## Data Availability

Datasets are not publicly available to protect participants' privacy but can be made available upon reasonable request.

## Appendix A Supplementary data

Supplementary S1 Participant flow and reasons for exclusion in the Ed.iTh trial. Supplementary S1
