# Supplementary material for: Online-CBT for endometriosis: Exploring the acceptability and impact of the Ed.iTh program
Source: Internet Interv. 2026 Jan 24;43:100906. doi: 10.1016/j.invent.2026.100906 (PMC12865585; doi:10.1016/j.invent.2026.100906)
Supplement: Supplementary S1 — Participant flow and reasons for exclusion in the Ed.iTh trial. [file mmc1.docx]

**Supplementary Material**

**S1. Participant flow and reasons for exclusion in the Ed.iTh trial.**

Of the 329 individuals expressing interest in the study, 64 did not proceed to telephone screening, due to being unreachable (*n* = 60) or declining participation (*n* = 4). The remaining 265 individuals underwent telephone screening. Of these, an additional seven individuals declined participation following screening, while 95 were excluded for not meeting eligibility criteria, including recent changes in hormonal treatment (*n* = 26), planned or current fertility treatments (*n* = 19), prior CBT for endometriosis within the last two years (*n* = 16), endometriosis not being the main burden (*n* = 13), previous hysterectomy (*n* = 13), presence of other severe physical illness (*n* = 6), regular intake of opioids (*n* = 1), and lack of laparoscopic confirmation of endometriosis (*n* = 1). In total, 163 participants were eligible and randomized in the primary trial.
